# The New Pelagic Operational Observatory of the Catalan Sea (OOCS) for the Multisensor Coordinated Measurement of Atmospheric and Oceanographic Conditions

**DOI:** 10.3390/s111211251

**Published:** 2011-11-28

**Authors:** Nixon Bahamon, Jacopo Aguzzi, Raffaele Bernardello, Miguel-Angel Ahumada-Sempoal, Joan Puigdefabregas, Jordi Cateura, Eduardo Muñoz, Zoila Velásquez, Antonio Cruzado

**Affiliations:** 1 Centre d’Estudis Avançats de Blanes (CEAB-CSIC), Carrer accés Cala St. Francesc 14, 17300 Blanes, Spain; E-Mail: tallercvb@gmail.com; 2 Instituto de Ciencias del Mar (ICM-CSIC), Passeig Marítim de la Barceloneta 37-49, 08003 Barcelona, Spain; E-Mail: jaguzzi@cmima.csic.es; 3 Department of Earth & Environmental Science, University of Pennsylvania, 240 S. 33rd Street, Hayden Hall 369, Philadelphia, PA 19104, USA; E-Mail: braf@sas.upenn.edu; 4 Universidad del Mar, Ciudad Universitaria s/n, Puerto Ángel, San Pedro Pochutla, 70902 Oaxaca, Mexico; E-Mail: ahumada@angel.umar.mx; 5 Laboratori d’Engynyeria Marítima, Universitat Politécnica de Catalunya, C/ Jordi Girona 1-3, Campus Nord-UPC, Edifici D1, 08034 Barcelona, Spain; E-Mails: joan.puigdefabregas@upc.edu (J.P.); jordi.cateura@upc.edu (J.C.); 6 Oceans Catalonia International SL, Anselm Clavé 8, 17300 Blanes, Spain; E-Mails: zoilavelasquez@gmail.com (Z.V.); acruzado@oceans.cat (A.C.)

**Keywords:** pelagic observatory, oceanographic buoy, multisensor coordinated monitoring, PAR, operational oceanography, submarine canyons, numerical multiparametric modelling, ocean forecast, Western Mediterranean Sea

## Abstract

The new pelagic Operational Observatory of the Catalan Sea (OOCS) for the coordinated multisensor measurement of atmospheric and oceanographic conditions has been recently installed (2009) in the Catalan Sea (41°39′N, 2°54′E; Western Mediterranean) and continuously operated (with minor maintenance gaps) until today. This multiparametric platform is moored at 192 m depth, 9.3 km off Blanes harbour (Girona, Spain). It is composed of a buoy holding atmospheric sensors and a set of oceanographic sensors measuring the water conditions over the upper 100 m depth. The station is located close to the head of the Blanes submarine canyon where an important multispecies pelagic and demersal fishery gives the station ecological and economic relevance. The OOCS provides important records on atmospheric and oceanographic conditions, the latter through the measurement of hydrological and biogeochemical parameters, at depths with a time resolution never attained before for this area of the Mediterranean. Twenty four moored sensors and probes operating in a coordinated fashion provide important data on Essential Ocean Variables (EOVs; UNESCO) such as temperature, salinity, pressure, dissolved oxygen, chlorophyll fluorescence, and turbidity. In comparison with other pelagic observatories presently operating in other world areas, OOCS also measures photosynthetic available radiation (PAR) from above the sea surface and at different depths in the upper 50 m. Data are recorded each 30 min and transmitted in real-time to a ground station via GPRS. This time series is published and automatically updated at the frequency of data collection on the official OOCS website (http://www.ceab.csic.es/~oceans). Under development are embedded automated routines for the *in situ* data treatment and assimilation into numerical models, in order to provide a reliable local marine processing forecast. In this work, our goal is to detail the OOCS multisensor architecture in relation to the coordinated capability for the remote, continuous and prolonged monitoring of atmospheric and oceanographic conditions, including data communication and storage. Accordingly, time series of measurements for a number of biological parameters will be presented for the summer months of 2011. Marine hindcast outputs from the numerical models implemented for simulating the conditions over the study area are shown. The strong changes of atmospheric conditions recorded in the last years over the area have altered the marine conditions of living organisms, but the dimension of the impact remains unclear. The OOCS multisensor coordinated monitoring has been specifically designed to address this issue, thus contributing to better understand the present environmental fluctuations and to provide a sound basis for a more accurate marine forecast system.

## 1. Introduction

Multisensor, remote, continuous and autonomous monitoring of the marine pelagic system (*i.e.*, water column) is essential in order to provide in real-time rigorous measurements of atmospheric and seawater conditions influencing the living resources [1,2]. Long-time series of biological and habitat data sets are required by governmental authorities and stakeholders for the management and conservation of important economic activities such as fishery [1,3,4]. Coordinated and multisensor measurement is presently pursued in order to take decisions on coastal maintenance and for the implementation of monitoring infrastructures that ultimately contribute to the sustainable management of coastal environments [2].

Around the world oceans, some permanent multisensor pelagic observatories such as the Bermuda-Atlantic Time Series Study (BATS) and the Hawaii Ocean Time Series (HOT) have been collecting data since 1988, thus providing long time-series of hydrographic and biogeochemical parameters, very useful for understanding local, regional, and global climate changes [3]. Those stations are presently a reference for research institutions carrying out multi-disciplinary studies on marine ecosystems functioning. In spite of the proven importance of such observing systems, to date few of them, such as those connected to the EuroSITES European Ocean Observatory Network (http://www.eurosites.info/) are functioning in the Mediterranean Sea. Among them, the DYFAMED station has been reporting hydrodynamic and biogeochemical data at an offshore station in the Ligurian Sea for more than 20 years. Other more recent stations in the Network providing diverse meteorological, chemical and physical parameters from the Mediterranean Sea are: W1-M3A, E1-M3A, E2-M3A, and ANTARES.

In the Catalan Sea coast (Spain), a network of Coastal Ocean Observatory stations is run by the Institute of Marine Sciences (ICM-CSIC; http://coo.icm.csic.es/) providing diverse marine and atmospheric information near shore. Another network of near-shore stations has been installed in the past decade by meteorological agencies of both the autonomous Catalan and Spanish governmental authorities, in order to provide meteorological and oceanographic data of relevance to commercial and recreational navigation (see: http://www.xiom.cat). Some research institutes and universities are also contributing to the acquisition of meteorological and oceanographic multiparametric habitat measurements in the coastal area. For example, the Technological Development Centre for the Remote Acquisition and Data Processing System (SARTI) installed in 2009 a permanent cabled multiparametric station in the Western Mediterranean area at 20 m depth, called Expandable Seafloor Observatory OBSEA [5]. Anyway, all these observing systems need improvements regarding the continuity and frequency of data acquisition and treatment, interconnection, analysis of the information collected and the real-time availability of resulting outputs. All these facts prevent their use for the formulation of more accurate oceanographic and climatological modelling, which would require decadal data sets in order to precisely depict the environmental variability at small and large basin scale area [6].

Recent calls on climate change management strategies address the need for implementing pelagic observatories, measuring the marine environment in a coordinated multisensor fashion. A set of environmental key variables, including photosynthetic available radiation (PAR; 400–700 nm), chlorophyll concentration (as a marker of phytoplankton biomass), dissolved oxygen and carbon dioxide, has been identified as Essential Ocean Variables (EOVs) [7]. These variables are not traditionally measured by oceanographic instrumentation in the observatories that generally measure only surface water temperature and currents (e.g., http://www.xiom.cat). The socio-economic interest of the EOVs recently moved the Scientific Committee for Oceanographic research (SCOR) of UNESCO to investigate for the current state of EOVs data in Spain [8]. The report indicates that in the Spanish coasts, most of data are collected near the seafloor (*i.e.*, benthic studies), whereas the pelagic zone still shows a limited number of physical and biogeochemical data.

The European Commission aims to consolidate ocean and marine observing systems in Europe (Brussels, 7.4.2009, SEC-2009, 499 Final Working Document; Commission of the European Communities). In this context, the new Operational Observatory of the Catalan Sea (OOCS) here presented is a pelagic multisensor platform installed in 2009 for the coordinated monitoring of the atmospheric and the oceanographic variables, including EOVs, at shallow depths (100 m) and medium time resolutions (30 min) not yet achieved anywhere else in the Mediterranean. So far, with funds provided by the Spanish Ministry of Science through the research project “Observation, Analysis and Modelling of the Mediterranean Sea”—OAMMS, ref. CTM2008-03983) the Observatory has been in operation for three years. Partial support will be provided in the near future by the Spanish Research Project entitled “Aerosol Deposition and Ocean Plankton Dynamics”—ADEPT (CTM2011-23458) approved to run up to Dec 2014 between 2012–2014 while funding sources from the local government operating several coastal buoys is also expected.

The data provided by multisensor pelagic observatories of this kind are a reference for assessing regional time changes of the marine conditions and for validating benthopelagic coupling models, which are being used to link climatic events to continental margin ecosystem responses [9]. For example, the multisensor OOCS system allows monitoring of chlorophyll variations in the upper 50 m in relation to temperature and light intensity, an aspect of great importance in the context of populations structure and distribution over sea floor gradients [10]. In this paper, our goal is to detail the architecture and coordinated functioning of OOCS sensors in relation to their capability for the long-time remote and continuous monitoring of atmospheric and oceanographic conditioning. Time series for different key habitat parameters, including EOVs are here presented. Data will be assimilated into numerical models presently under implementation, in order to show a feasible way to use OOCS data for atmospheric and marine forecasting.

## 2. The Observatory Architecture

### 2.1. The Study Area

**Figure 1.**
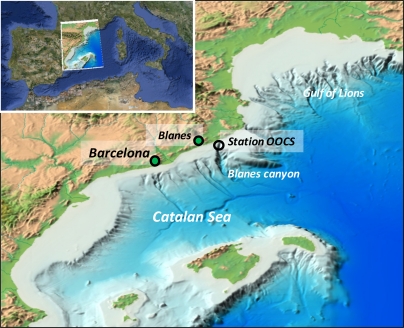
OOCS location in the Catalan Sea (western Mediterranean). The source of the Catalano-Balearic Sea-Bathymetric chart map is: www.icm.csic.es/geo/gma/MCB.

The OOCS was deployed in 2009 within the framework of the Spanish National Research Project “Observation, Analysis and Modelling of the Mediterranean Sea (OAMMS)” [11–13]. The OOCS observatory is composed of a fixed oceanographic buoy moored three miles offshore Blanes (Catalan Sea; 41°39.75′N, 02°54.57′E) at 192 m depth, in proximity of the shelf-break, on the Blanes canyon head (Figure 1). This canyon shows an axial bottom starting at 41°39′N, 2°54′E at 150 m depth and 5.4 km offshore, and extends southward perpendicularly to the coast. The axis reaches 1,000 m depth within 13 km and 1,500 m depth within 24 km [14]. Its topography deeply modifies the local sea circulation dominated by the Northern Current coming from the Gulf of Lions, producing cyclonic (anticlockwise) flow deflections around the shelf break in fall and summer [14,15].

The effects of canyon topography on the circulation and water properties have not yet been measured in any other similar area of the Mediterranean Sea with the time resolution provided by OOCS. Although the overall phytoplankton production in Blanes is low and a marked oligotrophic regime can be established for the pelagic environment [16], the canyon is known for its relatively high secondary production in relation to commercial extractive pelagic and demersal fishery [17,18]. Reports from local Fishermen’s Association indicate that bottom trawlers and longliners catch about 80 different species, mostly represented by European anchovy (*Engraulis encrasilocus*), red shrimp (*Aristeus antennatus*) and sardine (*Sardina pilchardus*) with variations of dominance depending on the season [http://www.actualrevista.net/].

### 2.2. Observatory Components

**Figure 2.**
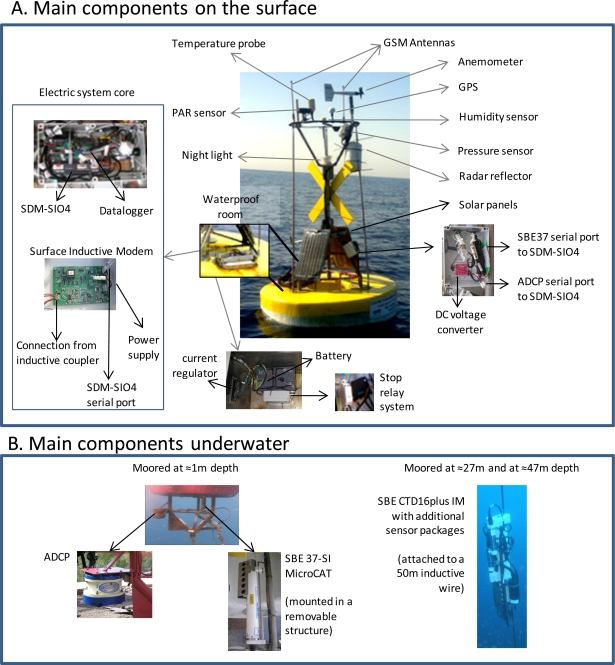
The OOCS key components in (**A**) the surface equipment and (**B**) underwater for the multisensor coordinated atmospheric and oceanographic monitoring (see Table 1 for details on installed sensors).

The buoy has a toroid-shaped surface unit, hosting various oceanographic and meteorological instruments along with two GSM modems for telemetry, a data logger for data storage, a GPS for correct positioning identification and finally, batteries charged by a set of photovoltaic panels through a charge controller (Figure 2). Connections among the system components are done with neoprene rubber and polyurethane-jacketed cables and Sub Conn connectors.

#### 2.2.1. The Buoy Structure

The float has a 2,000 mm diameter and 500 mm height, the flotation material consisting of dense polystyrene covered with high-resistance 10 mm fiberglass. A waterproof chamber for storing the battery, the datalogger and all additional electrical devices is installed inside the flotation material. The submerged structure, below the buoy, holds zinc anodes for cathode protection to avoid salt-water corrosion. 300 kg ballast hangs from the centre of the buoy keeping the buoy structure in a permanent upright position.

A single 2 m height mast, stainless steel made, holds all the meteorological instrumentation as well as a Garmin GPS and active and passive signalling for navigators in accordance with naval regulations. Two 45 watts solar photovoltaic panels provide battery charge during the sunlight hours and are sufficient for the shorter winter days showing a minimum of 8 h daylight. The rechargeable gel battery 12 V-70 Ah provides 5 amp powered by the two (2.5 amp) solar panels using a current regulator. The data acquisition system and control for the meteorological and oceanographic instrumentation installed both in the buoy structure and in the inductive mooring line is performed through a CR10X data logger. The batteries incorporate a stop relay system installed remotely reboot the system from the land station if required.

#### 2.2.2. The Mooring Line Configuration

**Figure 3.**
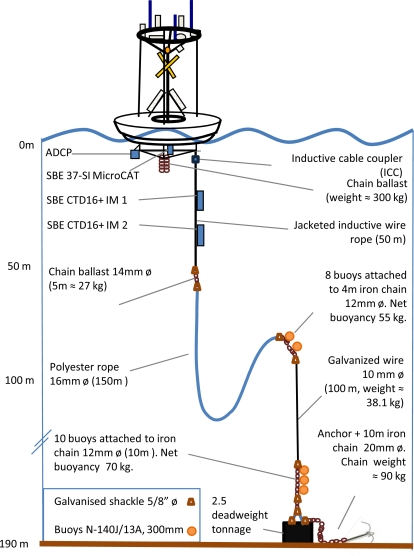
Inductive mooring configuration of the multisensory coordinated atmospheric and oceanographic monitoring.

The buoy is endowed with a mooring line anchored at 192 m depth (Figure 3). The upper part of that line is made up by a 50 m long inductive cable, holding two inductive CTD packs (Table 1), placed at 27 m and 47 m depth, respectively. An Inductive Cable Coupler (ICC) attached to the inductive cable about two meters below the buoy, is the underwater linkage between the inductive cable itself and the Surface Inductive Modem (SIM). The modem is stored in the waterproof case on the emerged side of the buoy (see Figure 2). The ICC creates a current flow travelling downward through the mooring cable, providing a voltage for the inductive CTDs data collection and transmission to the SIM. The lightweight polyester rope of the inductive cable provides high tensile strength (Figure 3). The galvanised wire connecting the mooring line to the seafloor provides robustness to the line, preventing any eventual break due to friction with the seabed. In total, the mooring line is 300 m, allowing the buoy and the 50 m inductive cable to be detached for maintenance and still leaving a mooring line length of 250 m length.

## 3. The Multisensor Coordinated Asset

The OOCS platform holds both atmospheric and oceanographic sensors, the latter deployed at different depths within the water column, down to 50 m. The oceanographic sensors record sea surface temperature, current speed and direction, dissolved oxygen, PAR, and fluorescence chlorophyll, which are declared EOVs (UNESCO). The 24 sensors and probes installed and their functioning specifications are presented in Table 1.

### 3.1. The Meteorological Sensors

The upper part of the 2,000 mm-height buoy mast holds instrumentation to measure wind speed and direction, as well as photosynthetically available radiation (PAR, 400–700 nm), air temperature, relative humidity and atmospheric pressure. A single jacketed cable containing all the cables from the meteorological instrumentation is guided downward through the mast up to the data-logger installed in the main waterproof case. Power supply (12 V) for all the meteorological instrumentation and the GPS is provided by the solar photovoltaic panels through a solar regulator installed in the main waterproof case installed in the float (see Figure 3).

### 3.2. The Oceanographic Sensors

The oceanographic instrumentation operates in different manners (see Figures 2 and 3). Just below the float, a SBE37 Microcat CT measures surface water temperature and salinity. The instrument is connected to the datalogger in the main waterproof case. A face-down RD Workhorse Monitor Acoustic Doppler Current Profiler (ADCP) is connected below the float. It is used to convert the backscattered sound into components of water current velocity in the upper 100 m layer. A 50 m length inductive mooring line holds at 27 m and 47 m depths two CTDs, SBE16 plus IM, inductively coupled to the mooring cable (see Table 1). The two SBE16 measure water temperature, salinity, pressure, and they have been coupled with other sensors for the measurement of different EOVs, such as dissolved oxygen, light intensity (PAR), turbidity, and chlorophyll fluorescence.

### 3.3. Management of Multisensor Data Collection, Transmission and Publication

**Figure 4.**
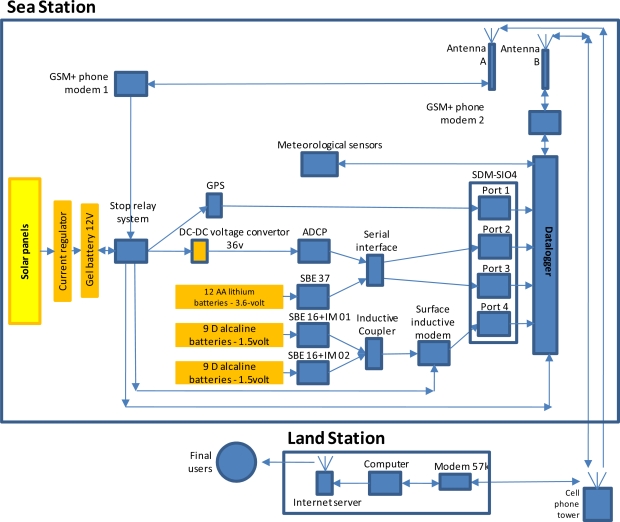
The flux diagram of the electric assemblage connecting the different sensors and powering sources of OOCS. This scheme is presented also in relation to the telemetry system used for data communication, storage and use at the land station.

The data acquisition system consists of a Campbell CR10X data-logger, installed in the waterproof case in the float (see Figure 2), collecting the data provided by all instrumentation in the buoy system. The datalogger runs a program (Loggernet) incorporating the calibration coefficients for all instruments. All the information collected is stored in a single text file within a flash memory card of the type lithium-backed Static Random Access Memory (SRAM) (see Figure 4). Scans rates of 5 s measure the meteorological conditions. The registered data are transmitted to the datalogger and recorded over a 30 min interval. There, the raw data are processed in order to obtain average values to be appended to the text file.

Regarding the oceanographic data acquisition, the SBE37 Microcat measures one data point every 30 min (no replicates are allowed from the system) after the pump runs for 20 s thus providing homogeneous water flux through the thermometer and conductimeter. The data are transmitted to the datalogger *via* cable and are appended to the text file, 10 minutes after recording. The two SBE 16 plus IM allow replicates, so that ten replicate measurements are taken after the pump runs for 20 s. The data are sent to the datalogger to process the data and to get (10 min later) the average values appended to the text file. Finally, the ADCP is set to correct pitch and roll for estimating current speed and direction at 22 cells starting from 7.1 m depth to a maximum range of 117.1 m depth, with a velocity standard deviation of 0.35 cm/s.

A SDM-SIO4 4-channel serial I/O interface is connected to the data-logger to provide serial port connections to the GPS (Port 1), ADCP (Port 2), SBE37 (Port 3), and the surface inductive modem SIM (Port 4) through four RS-232 serial ports. All the meteorological instrumentation connects directly to the datalogger, thus do not require serial interface system (see Figure 4).

The information retrieved is transmitted to the Centre for Advanced Studies of Blanes (CEAB) via mobile telephone call at hourly basis. A modem (GSM/GPRS) allows bidirectional communication for data and interrogation connections with the datalogger. Another modem allows unidirectional connection (from land to buoy) to remotely reboot the system in case of malfunction.

Data get published in real time at an official open-access web site (http://www.ceab.csic.es/~oceans/) by a self-activated Matlab software application, specifically designed for that purpose. The data file sent by the datalogger is composed of 118 columns. After appending the new data to the data file in the computer, the program executes automatically a routine to read the last collected string of 48 data (corresponding to the last 24 h) from the file and creates the plots sent through ftp connection to the CEAB’s server where the website allows public access to plots.

Alternatively, for the periods at which the buoy system is removed for maintenance or repair, nearly fortnightly profiles of the upper 100 m layer at the station are performed with a backup CTD and published at the website.

### 3.4. An Example of Real-Time Multisensor Coordinated Monitoring

Data collection at OOCS started in September 2009. Since this time, accidents, administrative issues, and programmed maintenance have forced several stops in the data acquisition. Therefore, presently available data sets are for the periods September 2009 to January 2010; April 2010; August 2010 to January 2011; April 2011 to May 2011; and finally, July 2011 to September 2011. In order to show the feasibility of OOCS to perform multisensor coordinated atmospheric and oceanographic monitoring in a continuous and long-lasting fashion, we present here 30 min data sets for the time interval between the 30 June and 24 August 2011.

**Figure 5.**
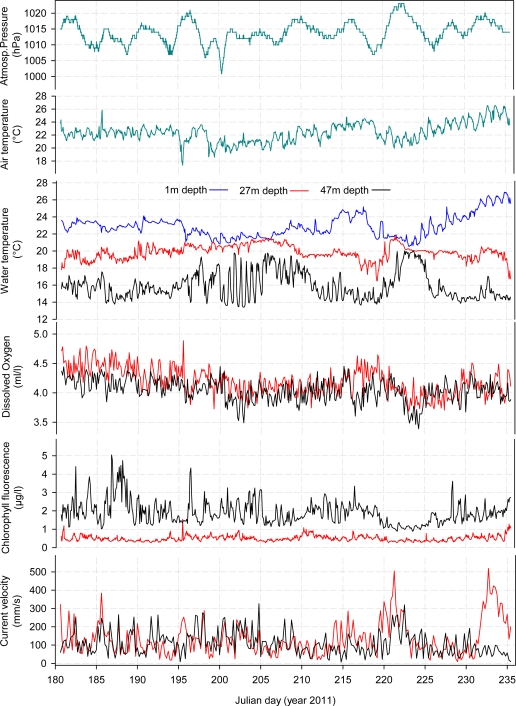
Time series of meteorological and oceanographic observations gathered in summer 2011 (*i.e.*, from 30 June to 24 August) at different depths (water surface at 1 m, at 27 m, and finally at 47 m depth).

From data acquired during this summer period it is possible to observe a strong diel variability of water temperature (Figure 5). Within the thermocline depth range, the water temperature shows changes of up to 4 °C within 12 h. This is visible with both inductive CTDs (*i.e.*, one at 27 m depth and another close to the boundary of the thermocline, at 47 m depth; see Table 1). This strong daily variation of water temperature has an important effect on the interpretation of classically collected oceanographic cruise data, covering station grid points over the Western Mediterranean area and showing strong hydrological variations. Misleading conclusions can be derived from unexpected fluctuations of water mass properties at a punctual time of sampling, as driven by processes such as internal wave propagation over the canyon bottom topography.

**Figure 6.**
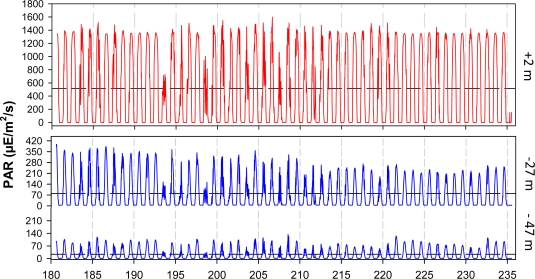
Photosynthetically Available Radiation (PAR; 400–700 nm) measured in the air and at the pelagic water layer above 50 m depth at the OOCS station in summer 2011 (*i.e.*, from 30 June to 24 August). The horizontal dashed line depicts the average measurement over the entire period of data acquisition (mean values are: 520 μE/m^2^/s at +2 m; 81 μE/m^2^/s at −27 m; and finally 18 μE/m^2^/s at −47 m).

The OOCS could efficiently measure light at diel and seasonal time scales as PAR at 2 m above the sea surface and both at 27 m and 47 m depths (Figure 6). Light sensors reported intensity variations over the 24-h scale at all depths, being the radiation reaching 27 m and 47 m depths the 16% and 6% respectively of the total radiation measured at 2 m above the sea surface. Light measurements showed daily variability as a result of cloudiness conditions. PAR is still neglected in most of marine ecological studies that therefore disregard the linkage between variations in biodiversity day-night behavioural rhythms of constituting species [19,20]. Also, PAR measurement is of importance within the framework of EOVs, being the primary driver of the ecosystem functioning, as it allows photosynthesis by phytoplankton transforming inorganic carbon (CO_2_) into organic matter (C_6_H_12_O_6_). Such matter will then be transferred to upper trophic levels such as fishes, crustaceans, marine mammals, *etc.*

**Figure 7.**
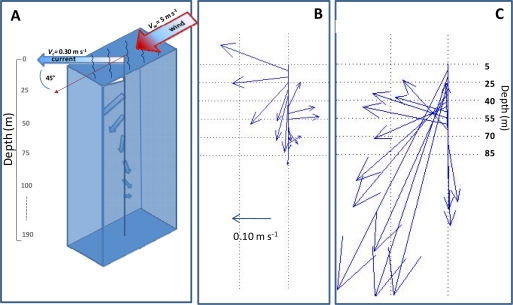
Approximation to a theoretical Ekman spiral from current data gathered at OOCS. In summer 2011 persistent strong S-E surface currents over the thermocline allowed approaching to an idealised Ekman spiral. Although the local currents were not driven by local winds (less than 2 m/s), the current intensities decreased with depth and formed a spiral similar to that of an idealised Ekman spiral. A, the theory suggests that a 5 m/s wind strength will promote a surface current velocity of about 0.30 m/s deviated 45% to the right at the latitude of the station location; B, the surface currents recorded at the station by the ADCP for 9 July 2011; and finally C, for 10 August 2011.

During the period of multiparametric data collection from 30 June to 24 August of the 2011, water currents were also sampled. Data sets showed the presence of strong summer surface currents flowing from the SE (Figure 7) running over the entire thermocline layer with about 45 m thickness. For instance, a steady current intensity from the SE of about 1 m/s took place in August 2011 lasting for about a week and allowing recording of a vertical pattern of marine currents similar to those of an Ekman spiral. The Ekman spiral theory suggests that a steady wind blowing over the surface ocean produces a surface current to about 45° to the right in the northern hemisphere. This current decreases exponentially with depth as its direction keeps rotating clockwise. Although the currents at the observation station did not decrease exponentially with depth (up to 80 m depth), the records indicated an approximation to the event, not easily measured in the real ocean [21].

### 3.5. Data Incorporation into Multiparametric Oceanographic Models

**Figure 8.**
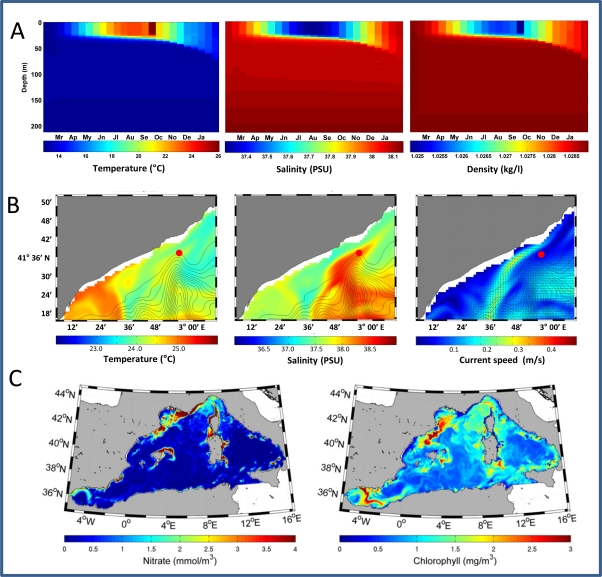
Examples of simulation outputs obtained assimilating OOCS data into numerical models operating at different space scales. (A) 1DV model simulations of water temperature, salinity, and density at the observation station for a whole year, imposing sea surface temperature and salinity provided by OOCS as boundary conditions; (B) simulations of high resolution sea surface temperature, salinity and velocity fields using the 3D model for the Blanes Canyon. The red circle over the canyon head indicates the location of the OOCS station; (C) simulations of sea surface nitrate concentration and chlorophylls concentration covering a wider western Mediterranean domain using the 3D model for the Western Mediterranean Sea.

Three numerical models of oceanographic forecast are currently under implementation within the framework of the OOCS data acquisition. A one-dimensional model with 3-m vertical resolution simulating the hydrological and biogeochemical conditions [16] is pursued in order to produce marine forecast through automated data assimilation. One-year model output incorporating average sea surface temperature from the periods at which the buoy has been deployed is shown as an example in Figure 8(A).

Additionally, two three-dimensional models simulating hydrodynamic and biogeochemical conditions of the Western Mediterranean Sea at different space resolutions are also presented as an example of potential larger scale forecast assimilating the *in-situ* data provided by the OOCS. Up to the present, these models have been used for hindcast simulations of the Mediterranean Sea conditions at different periods in the last decade. One of the models, at present under development, is a new high-resolution 3-D model simulating the circulation in the Blanes canyon and adjacent areas. The model has a 1/60° × 1/60° horizontal resolution and 32 sigma layers in the vertical and is nested in one-way off-line mode to a NW Mediterranean regional model (with 1/20° horizontal resolution and 32 sigma layers in the vertical) which in turn is nested to a basin-scale ocean circulation model called OPA (Océan PArallélisé), as a more General Mediterranean Forecasting System model [22–24], with 1/16° horizontal resolution and 72 unevenly z-levels in the vertical. Hindcast simulations of oceanographic events such as eddies around the canyon head along with the deflecting of the Northern Current in the vicinity of the canyon have been performed successfully (Figure 8(B)).

The last 3D model simulates the hydrodynamic and biogeochemical conditions of the Western Mediterranean Sea in the last decade, showing successful hindcast simulations of several EOVs such as sea surface temperature, chlorophyll concentration, bacterial biomass and dissolved inorganic nitrate [25]. The hydrodynamic module uses the Stony Brook Parallel Ocean Model (sbPOM) that is a parallel version of the Princeton Ocean Model (POM) [26]. The model domain includes the western Mediterranean Sea between 5.95W and 16.51E and between 34.97N and 44.52N. The western open lateral boundary is placed at the limit of the domain, on the Atlantic side of the strait of Gibraltar. The southern open lateral boundary is placed in the interior of the domain at 37.12N in the Sicilian Channel. The horizontal resolution is 1/20 degree so that the mesh size is constant with changes in longitude (5,560 m) but decreases northwards (from 4,456 m to 3,964 m). In the vertical dimension, the grid is resolved by 52 sigma-layers unevenly distributed with higher resolution at the surface and at the bottom. The simulations performed by the model suggest a complex effect of the hydrodynamics on the biogeochemical patters of EOVs (Figure 8(C)).

## 4. Discussion

In this study, we present a new pelagic multiparametric observatory measuring several important habitat parameters including those of ecological relevance for conservation (EOVs) from the water column and the atmosphere in a coordinated fashion. The technological implementation of this multisensor monitoring device occurred in the scenario of marine research where sensor innovation is rare. Few new sensor types are invented presently, with the exceptions of advancements in video-imaging techniques and supporting devices [27]. Most technological innovation in marine research concerns the coupling of different, already existing sensors, for air and sea measurements, the coordinated functioning and control representing the ultimate challenge [28]. Within this context, in this study we describe the architecture and functioning of a new pelagic observatory for the coordinated multisensor monitoring of the atmospheric and oceanographic western Mediterranean environment. We report data on several meteorological, biologic and oceanographic parameters among which there are important EOVs, presently neglected in the development of the majority of autonomous pelagic observatories to date installed in various continental margin areas of the planet [19,29]. This is the case of dissolved oxygen, chlorophyll concentration and associated PAR levels, as parameters of importance to monitor ocean productivity in spite of cyclically or stochastically variable climatologic conditions [3,6,25].

### 4.1. OOCS Potentials

Coastal observing systems are of great interest in high human impacted areas for monitoring seawater quality and the marine conditions [1,2]. The OOCS is located in a non-protected and highly impacted area. Local bottom trawlers and longliners have operated for more than 50 years over fishing grounds around the shelf-break and open slope of Blanes canyon, *i.e.*, fleets of bottom trawlers catching the red shrimp *A. antennatus* [17,18]. Other recreational activities are performed with particular intensity in the summertime, including sailing, kayaking and recreational fishing. Additionally, naval routes connecting several Mediterranean areas pass close to the coastline and nearby the observation station itself. In this context, real time data along with the numerical modelling of oceanographic and biogeochemical conditions in the canyon area and the entire western Mediterranean basin provide powerful tools for environmental authorities, coastal managers and marine service providers.

Several other features make the observatory different from other buoys and observatories in the area. The fixed observation station is located on the shelf-break (at 192 m depth), near the coastline (5.4 km offshore), what is a consequence of the narrow continental shelf along the *Costa Brava*. This means that the observatory is not a classical shallow coastal observatory with moorings e.g., at 20 m deep, but reflects the conditions over the continental shelf (above 200 m depth) and over the shelf-break. The shelf-break is the narrow continental shelf transition to the continental slope with a markedly increased slope gradient toward the canyon axis starting at about 150 m depth [14]. One of the main effects of the shelf-break is the deflection of the Northern Current along the canyon walls producing cyclonic eddies over the canyon head and upwelling of cooler and saltier water from about 200 m depth up to the lower base of the seasonal thermocline, located at about 45–50 m depth in the study area as deduced from OOCS observations [14]. The measurements at the Observatory (*i.e.*, currents, temperature, salinity) performed over the upper 100 m layer can record, at least partially, eventual changes produced in water properties due to deflection produced by the shelf-break.

The upwelling events are responsible for surface ocean fertilization eventually producing excess of organic matter accumulation [15]. Persistent fertilisation processes can jeopardize the survival of aerobic organisms, such as zooplankton and fish, because the bacterial organic matter oxidation process consumes large amounts of dissolved oxygen. That is the case of coastal upwelling areas over the western continental margins overseas and in coastal areas affected by excess nutrients (nitrate, phosphates), discharges from continental runoff produced by soil fertilisers [30]. Measuring dissolved oxygen in coastal areas is, therefore, of great utility to assess the water quality and to detect eventual signals of hypoxia, as demanded by international working teams on marine water quality [7]. Although no hypoxia events have been reported over the Catalan coast so far, the combination of an increasing water temperature and an excess of phytoplankton production could eventually lead the ecosystem toward unhealthy levels of hypoxia. At the observation station, no evidence of hypoxia events have been reported, even for periods of surface fertilization, the records always indicating dissolved oxygen values above 3.5 mL/L.

### 4.2. The Importance of Multiparametric Data Monitoring and Model Validation

The relatively complex hydrographic structure of the western Mediterranean and particularly the Catalan Sea influenced by the submarine canyons such as the Blanes canyon requires a better understanding. According to [31,32] the hydrographic features of the Catalan Sea area are influenced by: (a) the Atlantic water from Gibraltar surface inflow (temperature, T: 15–17 °C and salinity, S: 36.15–36.5); (b) the continental water from local river discharges (T-S variable depending on the fluxes and seasons, but always lower than in the surrounding waters); (c) the winter intermediate waters, formed in the Gulf of Lions(T: 12.5–13 °C and S: 38.1–38.3); (d) Levantine water from Sicilian strait inflow (T: 14–14.5 °C and S: 38.7–38.8); and finally, e) the winter Mediterranean deep water formed “locally” in wintertime (T 12.75–12.9 °C and S 38.4–38.48). Reports indicate overall increasing of surface, intermediate and deep water temperature and salinity all over western Mediterranean in last decades [33] in connection to strong seasonal changes of air temperature and precipitation regimes and overall temperature increasing and precipitation reduction [33,34] along with the increasing freshwater retention in reservoirs [34,35]. The changes in the atmospheric regimes over the western Mediterranean can even be locally opposing the adverse effects of Global Warming [34]. Nevertheless, the scarcity of available data and lack of long-time good quality measurements have prevented a better understanding (and modelling) of the complex air-sea interconnection and local responses to Global Warming [33–35].

Numerical modelling efforts are quite beneficial to understand space and time hydrographic complexity [33]. Here we show that data generated by OOCS and numerical ecological models contributes to understand local and mesoscale hydrographic properties (see Figures 7 and 8). Based on the hindcast simulations, the effects of environmental changes observed in last decades should be better understood and provide support for more accurate forecasting system for the area. To perform the forecast, the present numerical models will be adapted and will be used to assess the capability of the marine ecosystem response to environmental impacts. Presently, three numerical models are under implementation. First, the 1DV model simulating the oceanographic conditions in the stations assimilates temperature and salinity data from both the monthly CTD profiles and the continuous measurements for the periods that the buoy will be operative. A first approach to data assimilation has been reported here as an example of that forecasting capability (see Figure 8). Assimilation of biogeochemical properties, such as fluorescence chlorophyll and dissolved oxygen will follow in the near future. Second, the sea circulation over the Blanes canyon, including the observation station at the canyon head and extending the domain toward the whole canyon, is being simulated using a new high resolution 3D model. One of the oceanographic events to be addressed by the model is the effect of the canyon topography on the local circulation, namely the generation of cyclonic gyres by topographic steering of the North Current [14,15]. Furthermore, the presence of anticyclonic gyres is suggested from satellite observations and the complex spatial structure of the circulation over the canyon in the horizontal and vertical dimensions [14] requires 3D model simulations for better understanding the effect of the circulation on the organic and inorganic matter transport over the canyon [13]. Finally, a second 3D model (see Section 4.3) provides, at somewhat lower space resolution, the processes taking place over the western Mediterranean [13]. The first results demonstrate the model effectiveness in assessing the impact of the circulation over the organic and inorganic matter transport altering the primary production of phytoplankton and bacteria and secondary production of zooplankton and small pelagic organisms [21].

The Observatory is a promising setup for supporting scientific, academic, economic and ludic activities taking place within the highly impacted area by human activities in the Blanes canyon head. Preserving the observing system will make the meteorological and oceanographic time series, started in September 2009, to become longer and therefore to contribute to a better understanding of the short and long term environmental events produced within the coastal area, the shelf break and the Blanes canyon. Assimilation of collected multisensor coordinated measurements of atmospheric and oceanographic conditions into numerical models for providing further marine forecast will increase the Observatory operability and interest from the wide range of potential users.

### 4.3. Collaborative Scientific and Academic Activities and Dissemination Activities

One of the aims of OOCS is to establish connections with scientific and academic groups. Currently, the research project entitled “SMOS validation in the NW Mediterranean Sea” (SMOSCAL1.6862) is an international collaborative action lead by the European Space Agency (ESA) in relation to Mediterranean climate monitoring. The OOCS provides sea surface salinity and temperature data from the station location for supporting airborne campaigns aimed at measuring sea surface salinity using radiometers for calibration of the environmental satellite SMOS (Soil Moisture and Ocean Salinity). In return, OOCS benefits from direct access to SMOS/MIRAS Level 2 (Ocean salinity), ENVISAT MERIS L2 Full Res European Coverage NRT and recent—MER_FRS_2P (last 14 days) and AQUA-TERRA/ MODIS, Level1B European coverage.

Extensive scientific collaborations with research groups from the Institute of Marine Science (ICM-CSIC) in Barcelona are in progress. A collaborative action started in March 2010 with the aim of studying the vertical distribution of microbiological organisms, based on flow cytometer analyses at the OOCS (offshore) station and the Blanes Bay Microbial Observatory located off the port of Blanes. Collaboration with a research group from the Renewable Resources department at ICM has allowed reviewing the current operational cabled marine observatories around the world [5], performing and carrying out an oceanographic cruise on board the R/V Garcia del Cid aimed at studying the spatial variations of the physical properties along the Blanes canyon and evaluate the hydrographical effects on crustaceans spawns (work in progress). Finally, a new collaboration will start in January 2012 within the new funded Spanish Research Project ADEPT approved to run up to December 2014. The OOCS will provide oceanographic and meteorological data and will evaluate the relation with aerosol deposition.

The OOCS provide support to academic activities and short stays and visits from schools, graduate and postgraduate students at national and international level (e.g., the University of Barcelona, Spain; Fresenius University of Applied Sciences, Germany). Finally, the data and activities regarding the Observatory are a substantial component of the educational basis on marine observing systems and oceanography. The data produced by the Observatory are submitted to the consortium Mediterranean Operational Oceanography Network (MOON) and the National Oceanographic Data Center (NODC, NASA).

## Figures and Tables

**Table 1.** Meteorological and oceanographic sensors installed on the OOCS.

**Units**	**Sensor**	**Environmental variable**	**Main characteristics**	**Units**
*Meteorological instrumentation*				
1	107 Temperature Probe	Air Temperature	Measurement range: 0 to 70 °C. Accuracy: ±0.2 °C. Temperature measurement range: −35 to +50 °C	Celsius [°C]
1	CS100 Barometric Pressure Sensor	Atmospheric Pressure	Measurement range: 600 mb to 1,100 mb (hPa), −40 °C to +60 °C. Accuracy: ±0.03 mb	hPa
1	QSR-2000 Quantum Scalar Reference	Irradiance (PAR; 400–700 nm)	Measures sky irradiance over 400–700 μm (PAR)	μE/(cm^2^ × s)
1	HMP45C Temperature and Relative Humidity	Air Temperature	Measurement range: −40 °C to +60 °C. Accuracy at 20 °C ± 0.02 °C	Celsius [°C]
1		Relative Humidity	Measurement range: 0 to 100% non-condensing. Accuracy at 20 °C ± 2% RH (0 to 90% relative humidity)	%
1	05103 Young Wind Monitor	Wind speed	Measurement range: 0–134 mph (0–60 m s^−1^). Accuracy: ±0.6 mph (±0.3 m/ s)	m/s
		Wind direction	Measurement range: 0–360° mechanical, 355° electrical (5° open). Accuracy: ±3°	Degrees [°]
*Oceanographic instrumentation*				
1	SBE 37-SI MicroCAT CT	Water Temperature	Measurement range: −5 °C to 35 °C, Initial accuracy: 0.002 °C, Stability (per month): 0.0002 °C, precision: 0.0001 °C	Celsius [°C]
1		Water Conductivity (Salinity)	Measurement range: 0–7 S/m. Initial accuracy: 0.0003 S/m. Stability (per month): 0.00035 S/m. Precision: 0.00001 S/m	S/m
2	SBE CTD16plus IM	Water Temperature	Measurement range: −5 °C–35 °C, Initial accuracy: 0.005 °C. Stability (per month): 0.0002 °C. Resolution: 0.0001 °C	Celsius [°C]
2		Water Conductivity (Salinity)	Measurement range: 0–9 S/m. Initial accuracy: 0.0005 S/m. Stability (per month): 0.0003 S/m. Resolution: 0.00005 S/m.	PSU
2		Depth	Strain-gauge: 0 to 600. Initial accuracy: 0.1% of full scale range (FSR). Stability 0.004% FSR. Resolution 0.002% FSR.	dbar
2	Wetstar Fluorometer	Chlorophyll Fluorescence	Measurement range: 0 °C to 30 °C. Linearity ≥ 99% R^2^. Rresponse time: 0.125 s (digital). Accuracy: ≥0.03 μg/L. Dynamic ranges: 0.03–75 μg/L	μg/L
2	Biospherical Instruments Inc., QSP-2000, Quantum Scalar Sensor	PAR/Irradiance (Spherical collector with uniform directional response)	Measurement range: −2 °C–35 °C. Spectral response: ±10% quantum response (400–700 nm). Directional response: ±6% over all angles. Accuracy V = 1 × 1,017 quanta/(cm^2^ × s). Noise level: <1 mV	μE/(cm^2^ × s)
2	OBS Seapoint Turbidity Meter	Turbidity (Suspended Solids)	Output time constant: 0.1 s, RMS noise: <1 mV. Sensing distance <5 cm, Linearity: <2% deviation, Temp-coefficient: <0.05%/°C	FTU
2	Sea-Bird Dissolved Oxygen Sensor SBE43	Dissolved Oxygen	Measurement Range: 120% of surface saturation Initial accuracy: 2% of saturation. Typical stability: 0.5% per 1,000 h (clean membrane)	mL/L
1	RD Workhorse Monitor Acoustic Doppler Current Profiler—ADCP	Current Velocity (CV) & Current Direction (CD)	300 kHz. Vertical resolution range 126–95 m. SD 2 2.0 cm/s. Beam angle: 20°. Configuration: 4-beam, convex. Tilt: Range: ±15°, Compass Accuracy: ±2°. Precision: ±0.5°. Resolution: 0.01°. Maximum tilt: ±15°	CV: cm/s CD: Degrees [°]
1		Water Temperature	Temperature Measurement Range: −5 to 45 °C. Precision: ±0.4 °C. Accuracy: 0.01°	Celsius [°C]
